# Exploring the Role of Different Neonatal Nutrition Regimens during the First Week of Life by Urinary GC-MS Metabolomics

**DOI:** 10.3390/ijms17020265

**Published:** 2016-02-22

**Authors:** Angelica Dessì, Antonio Murgia, Rocco Agostino, Maria Grazia Pattumelli, Andrea Schirru, Paola Scano, Vassilios Fanos, Pierluigi Caboni

**Affiliations:** 1 Neonatal Intensive Care Unit, Puericulture Institute and Neonatal Section, Azienda Ospedaliera Universitaria, University of Cagliari, 09042 Monserrato, Italy; angelicadessi@hotmail.it (A.D.); andrea.schirru.as@gmail.com (A.S.); vafanos@tiscali.it (V.F.); 2 Department of Life and Environmental Sciences, University of Cagliari, Via Ospedale 72, 09124 Cagliari, Italy; a-murgia@hotmail.it; 3 Neonatal Intensive Unit and Neonatal Pathology, “S. Giovanni Calibita” Hospital, Fatebenefratelli Isola Tiberina, 00186 Rome, Italy; rocco.agostino@uniroma1.it (R.A.); mpattumelli@gmail.com (M.G.P.); 4 Department of Chemical and Geological Sciences, University of Cagliari, 09042 Monserrato, Italy; scano@unica.it

**Keywords:** breastfeeding, formula milk, metabonomics, IUGR, LGA, AGA, *myo*-inositol, glycine, pseudouridine

## Abstract

In this study, a gas-chromatography mass spectrometry (GC-MS) metabolomics study was applied to examine urine metabolite profiles of different classes of neonates under different nutrition regimens. The study population included 35 neonates, exclusively either breastfed or formula milk fed, in a seven-day timeframe. Urine samples were collected from intrauterine growth restriction (IUGR), large for gestational age (LGA), and appropriate gestational age (AGA) neonates. At birth, IUGR and LGA neonates showed similarities in their urine metabolite profiles that differed from AGA. When neonates started milk feeding, their metabolite excretion profile was strongly characterized by the different diet regimens. After three days of formula milk nutrition, urine had higher levels of glucose, galactose, glycine and *myo-*inositol, while up-regulated aconitic acid, aminomalonic acid and adipic acid were found in breast milk fed neonates. At seven days, neonates fed with formula milk shared higher levels of pseudouridine with IUGR and LGA at birth. Breastfed neonates shared up-regulated pyroglutamic acid, citric acid, and homoserine, with AGA at birth. The role of most important metabolites is herein discussed.

## 1. Introduction

Nutrition plays a key role in the life of every human being and can affect the quality of life in several ways. Nowadays, it is common knowledge that obesity and the risk to develop metabolic syndrome are correlated with either excessive or insufficient fetal nutrition [1,2]. It is also important how the nutrition in neonates could affect their future life quality or could prevent the risk of chronic pathologies. The “thrifty phenotype hypothesis”, by Hales *et al.* [3] proposes an association among low birth weight and increased risk in adulthood of developing diseases, helping to understand how to treat these patients and to reach new qualitative and valid standards in the field of infant nutrition.

Newborns are classified as adequate for the gestational age (AGA), when the weight is between the 10th and 90th percentile, while neonates with a weight over the 90th percentile are defined as large for gestational age (LGA), and those below the 10th percentile are small for the gestational age. Neonates with an intrauterine growth restriction (IUGR) have at birth a weight or a body mass index lower than normal for the number of gestational weeks [4]. Newborns belonging to the IUGR and LGA groups show a reduced carbohydrate tolerance with hypoglycemia [2]; this condition can lead in adulthood to a higher risk of metabolic diseases, such as type 2 diabetes and obesity. Besides intra-uterine quality of nutrition, diet in the first weeks of life can determine the future life of newborns. A valid alternative to the recommended breastfeeding is the use of formula milk, and to assess the effects in neonate metabolism is of primary importance in the field of nutrition. A noninvasive way of targeting this issue is the study of the metabolite urine excretion of formula milk (FM) and breastfed (BM) individuals. In this regard, a gas-chromatography mass spectrometry (GC-MS) based metabolomics has proven to be a good analytical tool candidate. Metabolomics is a new field of research, based on the quantitative analysis, over time, of the metabolic changes of a living system to pathological and physiological stimuli and genetic modifications [5]. This approach allowed evaluation of changes in neonate metabolism due to anthropometric parameters (IUGR and LGA) and nutrition [6,7,8,9,10,11,12,13]. The aim of the present study, based on a GC-MS metabolomics approach, was to ascertain whether and to what extent different diet regimens affect the urine metabolite profiles of IUGR, AGA and LGA neonates fed exclusively with either human breast milk (BM) or with formula milk (FM).

## 2. Results

**Figure 1:**
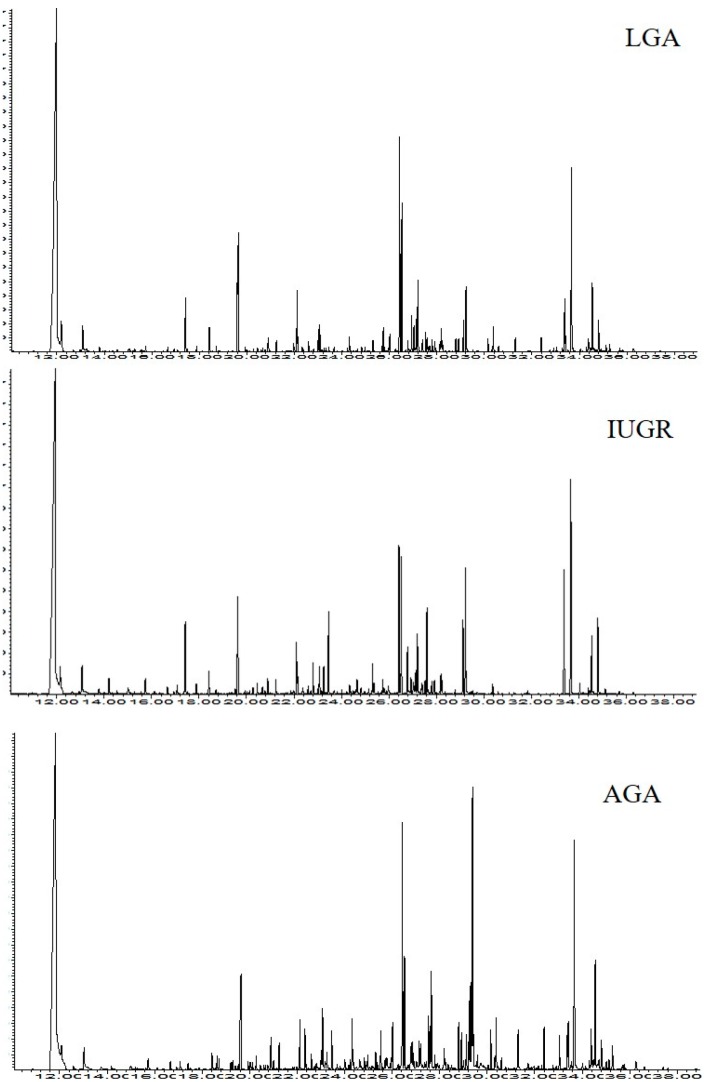
Gas-chromatography mass spectrometry (GC-MS) chromatograms of urine samples collected at the first day of life.

A total of 113 urine samples of AGA, IUGR and LGA neonates at T1 (at birth), T2 (three days) and T3 (seven days) were analysed by GC-MS. Representative chromatograms at T1 are shown in Figure 1.

From the analysis of the chromatograms, we observed 53 urine metabolites, such as short chain hydroxylated acids, long chain fatty acids, free amino acids, and mono- and disaccharides. Nineteen compounds were not identified and thereafter named U1–U19 (Table 1).

**Figure 2:**
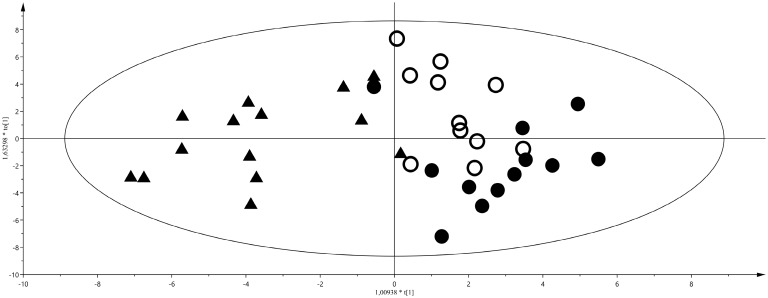
Orthogonal partial least square discriminant analysis (OPLS-DA) of appropriate gestational age (AGA) *vs.* intrauterine growth restriction (IUGR) + large for gestational age (LGA) neonates at birth, R^2^Y = 0.71, Q^2^Y = 0.36. Score plot, triangles represent the AGA class, filled and empty circles LGA and IUGR, respectively. Separation of classes is maximized through the predictive component (*x*-axis), intraclass variability is described through the orthogonal component (*y*-axis).

To investigate similarities and differences among samples on the basis of their metabolite profiles, a multivariate approach was applied. For this purpose, we created a matrix composed of 94 samples and 53 variables (metabolites). First, to study sample distribution and to detect outliers or common features, we ran a principal component analysis (PCA) for T1, T2 and T3. Surprisingly, no sample clusterization was observed on the basis of sex or neonate weight (data not shown). At T1, the metabolite profiles of IUGR and LGA samples overlapped. For this reason, we decided to group IUGR and LGA samples in a single class and to compare it to AGA samples by a pair-wise orthogonal partial least square discriminant analysis (OPLS-DA). This discriminant analysis demonstrated that, at birth, urine of IUGR and LGA neonates have a different metabolite profile than AGA neonates (see score plot in Figure 2; R^2^Y = 0.71, Q^2^Y = 0.36).

**Figure 3:**
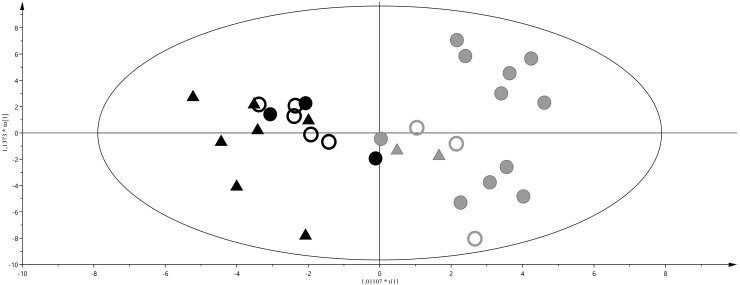
OPLS-DA of breast milk (BM) *vs.* formula milk (FM) fed neonates at T2, R^2^Y = 0.81 and Q^2^Y = 0.69. Score plot, triangles represent the AGA class, filled and empty circles LGA and IUGR, respectively. Black and grey colours represent BM and FM feeding, respectively. Separation of classes is maximized through the predictive component (*x*-axis), intraclass variability is described through the orthogonal component (*y*-axis).

From the analysis of the loading plot (data not shown) the most discriminating metabolites were: glucopyranoside, citric acid, and pyroglutamic acid for AGA, whilst threonic acid, pseudouridine and ribose for IUGR and LGA neonates. At T2 and T3, after feeding, IUGR and LGA cannot be differentiated from AGA. Therefore, we performed an OPLS-DA considering the urine of AGA, IUGR and LGA neonates at T2 and classifying them into two classes: breastfed and formula milk fed. A good discrimination was found between the two groups of neonates (R^2^Y = 0.81; Q^2^Y = 0.69) as shown in the score plot of Figure 3, with levels of glucose, galactose, and glycine higher in urine of neonates fed with FM, while aconitic acid, aminomalonic acid and adipic acid discriminated the BM diet.

**Figure 4:**
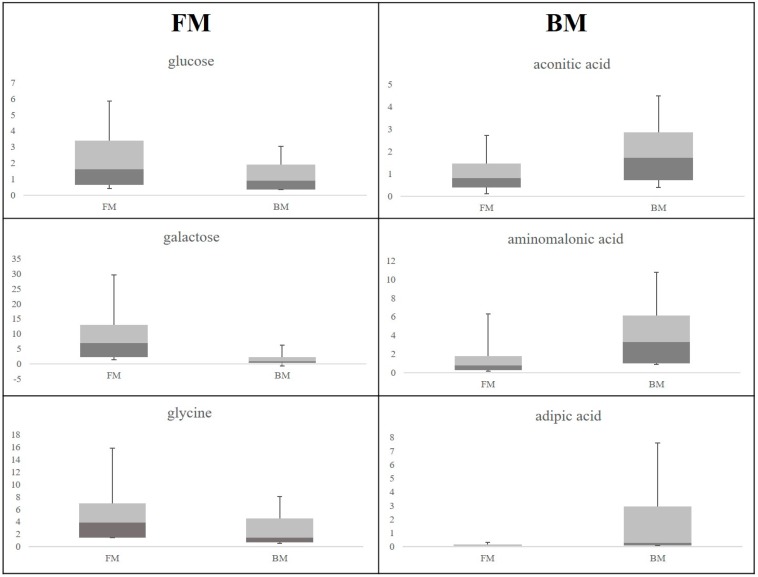
Comparison between the most discriminant metabolites. Box plots display the metabolite quantitative variation in each class.

For the two classes levels of these latter metabolites are reported in Figure 4. Other discriminant metabolites were *myo-*inositol, pseudouridine, citric acid and homoserine for FM, ribose for BM (data not shown).

**Figure 5:**
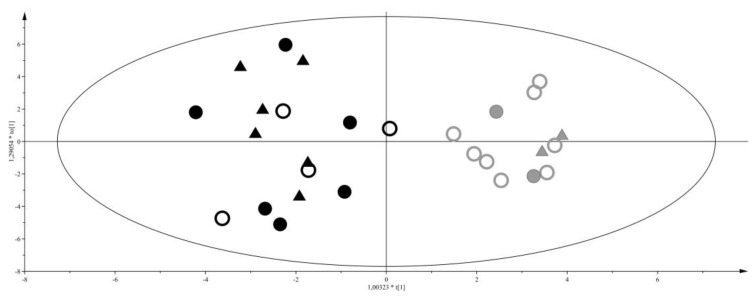
OPLS-DA of BM *vs.* FM neonates at T3, R^2^Y = 0.88 and Q^2^Y = 0.61. Score plot, triangles represent the AGA class, filled and empty circles LGA and IUGR, respectively. Black and grey colours represent BM and FM feeding, respectively.

The good performance of this model indicated a strong influence of diet in the urine metabolite profiles of neonates. A pair-wise OPLS-DA to the urine samples collected at T3 for BM *vs.* FM neonates was performed. This discriminant analysis (R^2^Y = 0.88, Q^2^Y = 0.61) indicated that also at T3 the diet strongly influenced urine metabolite profile. The score plot (Figure 5) shows that samples were correctly separated into two groups and that BM samples are more scattered than FM, highlighting in the former a higher intra-class variability.

**Figure 6:**
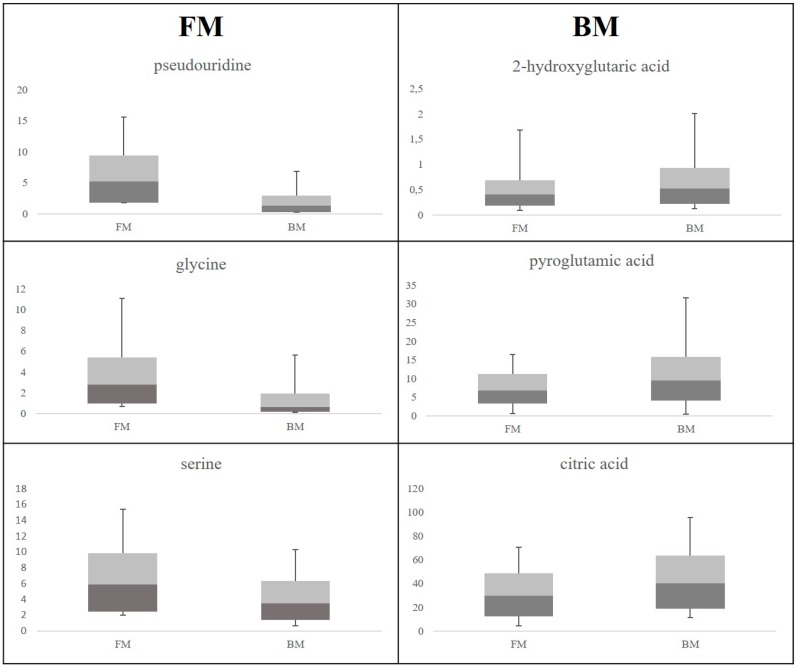
Comparison between most discriminant metabolites. Box plots display the metabolite quantitative variation in each class.

By the analysis of loadings, we selected the most discriminant metabolites reported as box plot in Figure 6: pseudouridine, glycine and serine were up-regulated in the FM class, while 2-hydroxyglutaric acid, pyroglutamic and citric acid were up-regulated in the BM class. Other discriminant metabolites were threonine and *myo*-inositol for the FM class, while ribose, homoserine and oxalic acid for the BM class (data not shown).

## 3. Discussion

### 3.1. Discriminant Metabolites for the Different Feeding Regimes

In this study, urine samples of newborns fed exclusively with BM or exclusively with FM at T2 and T3 were collected and analyzed. After the shift of nutrient supply from placenta to milk, the metabolic profile of IUGR, AGA and LGA newborns was more affected by diet than by their anthropometric parameters. At T2 and T3 breastfed babies showed different urine metabolite profiles compared to those FM fed. The discriminant metabolites reflect the metabolic interactions between the neonates and their diet. Specifically, at T2 the up-regulated metabolites in FM fed neonates were glucose, galactose, glycine, pseudouridine, citric acid, *myo*-inositol, and homoserine. The higher levels of glucose and galactose measured in FM compared with breast milk together with the higher intestinal permeability of FM fed neonates can explain the higher excretion of these sugars in FM fed neonates [14]. At T2, urine of neonates fed BM showed higher levels of aconitic acid, aminomalonic acid and adipic acid when compared to FM. The higher levels of adipic acid, probably reflect an elevated lipolysis and augmented catabolism of fatty acids in these neonates to compensate the low energy level of colostrum [15,16]. This hypothesis can be also valid for aconitic acid that is a Krebs cycle compound. Aminomalonic acid is an amino dicarboxylic acid derived from cysteine and marker of oxidative stress [17].

At T3, discriminant metabolites for FM fed were pseudouridine, glycine, serine, threonine, and *myo*-inositol. Noteworthy, newborns fed with FM shared the same representative metabolites (pseudouridine, *myo*-inositol and glycine) with IUGR and LGA neonates at birth, which raises questions about the quality of FM, especially when compared with BM. The benefits of breastfeeding are a topic of interest in medicine, involving different fields, from gynecology to pediatrics [18]. It is well known that breastfeeding affects both mother and child positively [19,20]. BM neonates were higher in pyroglutamic acid, a compound of the glutathione cycle, found up-regulated in AGA neonates at birth. Friesen *et al.* [21] measured the plasma pyroglutamic acid levels in pregnant women and in their newborns finding that pyroglutamic acid was 56% elevated in pregnant women, and with higher levels in fetal plasma compared with maternal plasma [22]. Jackson A. *et al.* [23] reported that, when compared with adults, neonates excrete five to ten times the amount of pyroglutamic acid.

### 3.2. Maternal Malnutrition and Consequences on Neonates’ Health

Several events may occur during intrauterine life, which could influence the future life of the fetus. In the case of IUGR neonates, maternal malnutrition, smoking and placental vascular disease are the main causes for not reaching full growth potential, with a lower body mass index and weight for the number of gestational weeks [24,25]. On the other hand, macrosomia is characterized by an increased body mass index and weight above the 90th percentile for gestational age. The main risk factors are: maternal diabetes, obesity and nutrition/weight gain during pregnancy [26].

Both fetal hyponutrition (IUGR) and hypernutrition (LGA) at birth can favor the developing of a low carbohydrate tolerance due to hypoglycemia. These findings can be strengthened by results of our study, in fact, the analysis of the samples collected in the first day of life revealed similarities between IUGR and LGA neonates when compared to AGA neonates, LGA and IUGR sharing the same up-regulated metabolites: threonic acid, pseudouridine, ribose, and, to a lesser extent, *myo*-inositol, and glycine. Lawson *et al.* [27] reported that erythronic acid and threonic acid urine excretion was altered in adult individuals fed with synthetic diet rich of high carbohydrates.

In our statistical model, LGA and IUGR neonates at birth showed up-regulated threonic acid suggesting an altered carbohydrates metabolism. Recently, d-ribose has been found to be present at an abnormally high concentration in the urine of type 2 diabetics, suggesting a key role in metabolism carbohydrates disorders [28]. Su *et al.* [28] demonstrated that mouse spatial cognitive impairment is caused by ribose derived advanced glycation end products, partially proving a risk factor for IUGR neonates’ cognitive dysfunction [29,30]. Higher levels of glycine and *myo*-inositol in IUGR and LGA neonates were already observed in our recent works [31,32]. The occurrence of *myo*-inositol in the urine is in accordance with the reduced carbohydrate tolerance due to the hypoglycemic and hyperglycemic status of IUGR and LGA neonates during the fetal life, and it should be considered as a marker of an altered glucose metabolism during fetal development [33]. Moreover, newborns consume glycine to produce purines and heme, providing one third of the residues for the formation of collagen and elastin [34], and is produced by the placenta in large amounts [23,26]. Gannon *et al.* [35] have demonstrated that the ingestion of glycine induced an increase of glucagon levels. Furthermore, when glycine is ingested with glucose, the insulin peak occurs later and is lower if compared to when glucose is ingested alone [35].

Glycine plays also a key role in the synthesis of the glutathione (GSH), which is an intracellular scavenger of free radicals and reactive oxygen species. Numerous diseases in premature neonates, such as bronchopulmonary dysplasia, periventricular leukomalacia, intraventricular hemorrhage, retinopathy of prematurity and necrotizing enterocolitis have been associated to oxidative stress, decreased GSH, and free radical-mediated tissue injury [36,37,38,39,40]. In presence of diabetes or high glucagon levels, the oxidation of glycine in hepatic cells increases and it may become a limiting factor in the synthesis of GSH [41]. Different studies showed that glycine availability is able to limit erythrocyte GSH synthesis in burn patients [42] or in malnourished children [43]. Up-regulated urine levels of glycine in both IUGR and LGA neonates after birth may reflect the altered metabolic status of these infants, which appears to be predominantly catabolic, with a potential oxidative stress damage. Pseudouridine is an atypical nucleoside found in human urine as an RNA metabolite, specifically the degradation rate of rRNA was determined through the excretion of pseudouridine. Literature data suggest that the excretion rate of pseudouridine in healthy people is a valid non-invasive indicator of resting metabolic rate [44]. From the evidence that pseudouridine inhibits basal glucose consumption in rat soleus muscle, Dzúrik *et al.* [45] concluded that pseudouridine inhibits glucose use in the calcium modulation of the insulin regulatory cascade.

In the metabolic profile of AGA neonates, representative metabolites were glucopyranoside and organic acids such as citric acid, and benzoic acid. It has been postulated that urinary low molecular weight organic acid excretion in the neonate, which differs from the adult urinary pattern, suggests a reflection of the particular neonatal metabolism, including a high fatty acid use and a low protein catabolism [16].

## 4. Experimental Section

### 4.1. Subjects

This study was carried out on 113 urine samples from patients admitted to the Neonatal Intensive Care Unit, Puericulture Institute and Neonatal Section, Azienda Ospedaliera Universitaria, University of Cagliari (Cagliari, Italy) and to the Neonatal Intensive Unit and Neonatal Pathology of “S. Giovanni Calibita” Hospital Fatebenefratelli (Rome, Italy). The study protocol was approved by the local ethical committee and written informed consent was obtained from the parents before enrolment in the study (CA-206-18/03/2013). According to the anthropometric classification, neonates were divided in three groups: (1) AGA, *n* = 14 of which 6/8 males/females, mean gestational age 38.3 weeks, mean birth-weight (BW) 3033 ± 287 g (percentiles = 49.2); (2) IUGR, *n* = 10, 5/5 m/f, mean gestational age of 37 weeks, mean BW 2313 ± 365 g (percentiles = 6.9); (3) LGA, *n* = 11, 5/6 m/f, mean gestational age of 38.6 weeks, mean BW of 3858 ± 313 g (percentiles = 94.3). Urine samples were matched for sex, age, gestation and type of delivery, and were collected at different times: within 8 h of birth prior to the administration of food (first urine output) (T1), at the third day of life (T2) and at the seventh day of life (T3). The total number of samples collected was 31 for AGA (T1 = 14; T2 = 9; T3 = 8), 30 for IUGR (T1 = 10; T2 = 8; T3 = 12) and 33 (T1 = 11; T2 = 14; T3 = 8) for LGA. From T2, data regarding the infants’ feeding regime were also collected, selecting only neonates fed exclusively with either FM or BM and excluding infants with mixed feeding (*n* = 19). At T2, 15 neonates of the studied population were fed with BM (7 AGA, 3 LGA, 5 IUGR) and 16 with FM (2 AGA, 11 LGA, 3 IUGR). At T3, 16 neonates were fed with BM (6 AGA, 6 LGA, 4 IUGR) and 12 with FM (2 AGA, 2 LGA, 8 IUGR). All the newborns survived. The clinical data of each patient was recorded in the hospital registers.

### 4.2. Chemicals

Chemicals, analytical standards and solvents were purchased from (Sigma Aldrich, Milano, Italy). Derivatized 2,2,3,3-d4-succinic acid was used as internal standard. Methoxyamine hydrochloride, *N*,*O*-bis(trimethylsilyl)trifluoroacetamide, trimethylchlorosilane (BSTFA + TMCS) were used for derivatization purposes.

### 4.3. Urine Samples Collection and Preparation

Urine samples (2–3 mL) were collected using a non-invasive method with a ball of cotton, then aspirated with a syringe and transferred to a sterile 15 mL Falcon tube. The tubes were then stored at −80 °C. GC-MS, analyses were carried out at the Department of Life and Environmental Sciences laboratories in Cagliari. After thawing on ice, the urine samples were centrifuged for 10 min at 13,200 rpm. One hundred and fifty μL of supernatant from each sample were transferred to Eppendorf tubes containing 1 mg of urease, sonicated for 30 min at 37 °C and then centrifuged for 10 min at 13,200 rpm. The supernatant was separated and after drying derivatized with 50 μL of methoxyamine in pyridine solution (10 mg/mL). After 17 h, 100 μL of BSTFA + TMCS were added and after 1 h samples were resuspended with 600 μL of hexane. Trimethylsylilated 2,2,3,3-d4-succinic acid at 5 mg/L was used as internal standard.

### 4.4. GC-MS Analysis

Analysis was conducted injecting one microliter of derivatized sample in a 6850 gas chromatograph attached with a 5973 Network mass spectrometer (Agilent Technologies, Santa Clara, CA, USA). GC separation was obtained with a 30 m × 0.25 mm ID, silica chemically bonded capillary column (J&W Scientific, Folsom, CA, USA). The temperature of the injector was 200 °C and the gas flow rate was 1 mL/min. The column initial temperature was mantained at 50 °C for 10 min and then increased to 300 °C at 10 °C/min and held at 300 °C for 10 min. The transfer line and the ion source temperatures were at 280 and 180 °C, respectively. Ions produced at 70 eV were recorded at 1.6 scan/s in the mass range *m*/*z* 50–550. GC-MS data analysis was conducted by integrating each resolved chromatogram peak and normalizing the area for the corrected total area of the chromatogram. Peaks were examined for their mass spectra and their identification was performed using the NIST08 library after deconvolution with an automated mass spectral deconvolution and identification system (AMDIS).

### 4.5. Multivariate Data Analysis

A multivariate data analysis was performed using the SIMCA software (version 13.0, Umetrics, Umea, Sweden). Untargeted principal components analysis (PCA) was applied to visualize tendency of samples to cluster on the basis of similarities in their metabolite profile and to detect the presence of outliers. The latter can be detected by different tests, as implemented in SIMCA software. For sample classification and for the search of biomarkers that differentiate the predefined classes, the Orthogonal Partial Least Square-Discriminant Analysis (OPLS-DA) was performed, this is a supervised classification method that requires information about class membership of samples. In OPLS-DA, class separation is maximized in the predictive component (*x*-axis) and its orthogonal component (*y*-axis) express intra-class variability. The goodness of models was evaluated by the parameters: 3R^2^Y (goodness of classification) and Q^2^Y (goodness of classification in cross-validation) [46].

## 5. Conclusions

In summary, our results show that at birth the urinary metabolome of AGA neonates differs from that of IUGR and LGA neonates, and that, when the nutrition supply moves from intrauterine to lactation or bottle feeding, the typology of milk (breast or formula) strongly characterize the urine metabolite profiles of neonates overcoming anthropometric classification. This confirmed the importance of quality of nutrition in the first days of life and also proves that this GC-MS based metabolomics approach is very sensitive to diet regimens and therefore endorses it as a helpful efficient tool in the research field of neonatal feeding. While published studies exist on milk metabolomics, this study, to our knowledge, the first report in literature, focuses on the different metabolic impacts that breast milk and formula milk display in infants of different categories (IUGR, AGA, LGA) in the first week of life. These data and ongoing studies on this topic may be very important from a practical point of view in selecting the formula milks that not only have physicochemical properties more similar to breast milk, but also determine metabolic and nutritional impacts similar to breast milk. We want to highlight the importance for neonates to consume breast milk when possible, and the need for the development of formula milk to more closely emulate breast milk. Future perinatal studies should also take into account an integration with maternal metabolic data.

## Figures and Tables

**Table 1:** Gas-chromatography mass spectrometry (GC-MS) characteristics of urine metabolites.

Compound	Rt (min)	EI-MS, *m*/*z* (amu) (% Relative Abundance)	Trivial Name	Abbr
Alanine	16.72	116 (100), 73 (50), 147 (30)	alanine	ALA
Glycine (2 TMS)	17.14	102 (100), 73 (50), 147 (45)	glycine (2 TMS)	GLY2
Oxalic acid	17.48	147 (100), 73 (80), 133 (50)	oxalic acid	OXA
3-Hydroxybutanoic acid	17.86	147 (100), 73 (50), 117 (45)	3-hydroxybutyric acid	3HBA
Benzoic acid	19.36	179 (100), 105 (64), 135 (55)	benzoic acid	BA
Glycine (3 TMS)	20.23	174 (100), 73 (50), 248 (47)	glycine (3 TMS)	GLY3
Butanedioic acid	20.37	147 (100), 73 (50), 247 (45)	succinic acid	BUA
2,3-Dihydroxypropanoic acid	20.54	171 (100), 100 (50), 292 (45)	glyceric acid	GLYA
2,3-Dihydroxybutanoic acid	20.72	73 (100), 292 (50), 147 (45)	2,3-dihydroxybutyric acid	DHB
Serine	20.99	204 (100), 73 (70), 218 (65)	serine	SER
Threonine	21.33	73 (100), 218 (94), 291 (50)	threonine	THR
Unknown 1	21.70	103 (100), 73 (90), 219 (60)	U1	
3,4-Dihydroxybutanoic acid	21.97	73 (100), 233 (60), 147 (50)	2-deoxytetronic acid	DOT A
Homoserine	22.21	218 (100), 73 (50), 128 (50)	homoserine	HOMO
Aminomalonic acid	22.49	147 (100), 73 (95), 218 (90)	aminomalonic acid	AMIN A
Unknown 2	22.57	158 (100), 68 (50), 147 (45)	U2	
Unknown 3	22.92	73 (100), 217 (90), 147 (80)	U3	
Hexanedioic acid	22.99	73 (100), 111 (68), 147 (50)	adipic acid	HA
Aspartic acid	23.12	232 (100), 73 (90), 100 (40)	aspartic acid	ASP A
5-Oxo-1-proline	23.19	156 (100), 73 (50), 147(40)	pyroglutamic acid	PYR A
2,3,4-Trihydroxybutanoic acid	23.39	73 (100), 292 (90), 147 (50)	threonic acid	THR A
2-Hydroxypentanedioic acid	23.80	129 (100), 147 (90), 247 (80)	2-hydroxyglutaric acid	HGLU A
Unknown 4	24.34	73 (100), 231 (90), 147 (43)	U4	
Phenylalanine	24.46	333 (100), 73 (80), 218 (40)	phenylalanine	PHEN
Unknown 5	24.50	73 (100), 200 (80), 267 (70)	U5	
Unknown 6	24.79	73 (100), 103 (60), 217 (50)	U6	
Ribose	24.94	73 (100), 205 (50), 147 (40)	ribose	RIB
Unknown 7	25.08	73 (100), 147 (90), 247 (70)	U7	
Xylitol	25.44	73 (100), 217 (90), 307 (55)	xylitol	XYL
Fucose	25.5	117 (100), 73 (90), 217 (76)	fucose	FUC
Unknown 8	25.67	73 (100), 117 (90), 160 (30)	U8	
1-Propene-1,2,3-tricarboxylic acid	25.83	73 (100), 147 (90), 229 (50)	aconitic acid	ACO A
Unknown 9	25.93	73 (100), 292 (70), 357 (40)	U9	
1,2,3-Propanetricarboxylic acid	26.58	273 (100), 147 (60), 73 (60)	citric acid	CIT A
Unknown 10	26.93	73 (100), 217 (92), 147 (70)	U10	
Galactose	27.29	73 (100), 319 (90), 205 (60)	galactose	GAL
Glucose	27.52	73 (100), 319 (72), 205 (54)	glucose	GLU
Unknown 11	27.75	333 (100), 73 (58), 160 (43)	U11	
Unknown 12	28.04	373 (100), 73 (90), 358 (70)	U12	
Hexadecanoic acid	28.95	313 (100), 117 (90), 73 (90)	palmitic acid	HEX A
Unknown 13	29.16	73 (100), 103 (43), 244 (28)	U13	
*Myo*-inositol	29.29	305 (100), 217 (90), 73 (53)	myoinositol	MYO
Octadecanoic acid	30.75	341 (100), 205 (98), 117 (76)	stearic acid	OCT A
Pseudouridine	31.43	217 (100), 73 (50), 357 (40)	pseudouridine	PURID
Unknonwn 14	31.87	73 (100), 217 (50), 246 (49)	U14	
Unknonwn 15	32.01	217 (100), 73 (55), 147 (40)	U15	
Unknonwn 16	32.44	285 (100), 73 (55), 186 (40)	U16	
2,3-Dihydroxy hexadecanoic acid	33.52	371 (100), 73 (40), 147 (40)	2,3-dihydroxyhexadecanoic acid	DHH A
Glucopyranoside	33.82	217 (100), 73 (90), 147 (60)	glucopyranoside	GPYR
Unknonwn 17	34.21	204 (100), 73 (90), 361 (80)	U17	
Unknonwn 18	34.33	204 (100), 73 (90), 361 (80)	U18	
Unknonwn 19	34.71	204 (100), 73 (70), 361 (62)	U19	
2,3-Dihydroxy octadecanoic acid	34.95	73 (100), 191 (90), 361 (70)	2,3-dihydroxy octadecanoic acid	DHO A

TMS = trimethylsilyl derivative.

## References

[1] Barker D.J. (1995). Fetal origins of coronary heart disease. BMJ.

[2] Dessì A., Puddu M., Ottonello G., Fanos V. (2013). Metabolomics and fetal-neonatal nutrition: Between “not enough” and “too much”. Molecules.

[3] Hales C.N., Barker D.J. (1992). Type 2 (non-insulin-dependent) diabetes mellitus: The thrifty phenotype hypothesis. Diabetologia.

[4] Committee on Practice Bulletins Gynecology, American College of Obstetricians and Gynecologists (2001). Intrauterine growth restriction. Clinical management guidelines for obstetrician-gynecologists. Int. J. Gynaecol. Obstet..

[5] Nicholson J.K., Connelly J., Lindon J.C., Holmes E. (2002). Metabonomics: A platform for studying drug toxicity and gene function. Nat. Rev. Drug Discov..

[6] Dessì A., Pravettoni C., Cesare Marincola F., Schirru A., Fanos V. (2015). The biomarkers of fetal growth in intrauterine growth retardation and large for gestational age cases: From adipocytokines to a metabolomic all-in-one tool. Expert Rev. Proteom..

[7] Dessì A., Cesare Marincola F., Masili A., Gazzolo D., Fanos V. (2014). Clinical metabolomics and nutrition: The new frontier in neonatology and pediatrics. BioMed Res. Int..

[8] Marincola F.C., Noto A., Caboni P., Reali A., Barberini L., Lussu M., Murgia F., Santoru M.L., Atzori L., Fanos V. (2012). Metabolomic study of preterm human and formula milk by high resolution NMR and GC/MS analysis: Preliminary results. J. Matern. Fetal Neonatal Med..

[9] Dessì A., Atzori L., Noto A., Visser G.H.A., Gazzolo D., Zanardo V., Barberini L., Puddu M., Ottonello G., Atzei A. (2011). Metabolomics in newborns with intrauterine growth retardation (IUGR): Urine reveals markers of metabolic syndrome. J. Matern. Fetal Neonatal Med..

[10] Horgan R.P., Broadhurst D.I., Dunn W.B., Brown M., Heazell A.E.P., Kell D.B., Baker P.N., Kenny L.C. (2010). Changes in the metabolic footprint of placental explant-conditioned medium cultured in different oxygen tensions from placentas of small for gestational age and normal pregnancies. Placenta.

[11] Logan K.M., Wijeyesekera A.D., Perez I.G., Hyde M.J., Romero M.G., Jeffries S., Andreas N., Gale C., Holmes E., Modi N. Infants of Mothers with Diabetes Have Altered Urinary Metabolic Profile at Birth. Proceedings of the Neonatal Society 2012 Summer Meeting.

[12] Favretto D., Cosmi E., Ragazzi E., Visentin S., Tucci M., Fais P., Cecchetto G., Zanardo V., Viel G., Ferrara S.D. (2012). Cord blood metabolomic profiling in intrauterine growth restriction. Anal. Bioanal. Chem..

[13] Dessì A., Marincola F.C., Fanos V. (2015). Metabolomics and the great obstetrical syndromes—GDM, PET, and IUGR. Best Pract. Res. Clin. Obstet. Gynaecol..

[14] Taylor S.N., Basile L.A., Ebeling M., Wagner C.L. (2009). Intestinal permeability in preterm infants by feeding type: Mother’s milk *versus* formula. Breastfeed. Med..

[15] Alm J., Hagenfeldt L., Larsson A. (1978). Concentrations of organic acids in the urine of healthy newborn children. Ann. Clin. Biochem..

[16] Gregersen N., Ingerslev J., Rasmussen K. (1977). Low molecular weight organic acids in the urine of the newborn. Acta Paediatr. Scand..

[17] Ibarra R., Dazard J.E., Sandlers Y., Rehman F., Abbas R., Kombu R., Zhang G.F., Brunengraber H., Sanabria J. (2014). Metabolomic analysis of liver tissue from the VX2 rabbit model of secondary liver tumors. HPB Surg..

[18] Gates S.C., Sweeley C.C., KrIvIt W., DeWitt D., Blaisdell B.E. (1978). Automated metabolic profiling of organic acids in human urine. II. Analysis of urine samples from “healthy” adults, sick children, and children with neuroblastoma. Clin. Chem..

[19] Marincola F.C., Dessì A., Corbu S., Reali A., Fanos V. (2015). Clinical impact of human breast milk metabolomics. Clin. Chim. Acta.

[20] Labbok M.H. (2001). Effects of breastfeeding on the mother. Pediatr. Clin. N. Am..

[21] Friesen R.W., Novak E.M., Hasman D., Innis S.M. (2007). Relationship of dimethylglycine, choline, and betaine with oxoproline in plasma of pregnant women and their newborn infants. J. Nutr..

[22] Martin R.M., Gunnell D., Smith G.D. (2005). Breastfeeding in infancy and blood pressure in later life: Systematic review and meta-analysis. Am. J. Epidemiol..

[23] Jackson A.A., Persaud C., Hall M., Smith S. (1997). Urinary excretion of 5-l-oxoproline (pyroglutamic acid) during early life in term and preterm infants. Arch. Dis. Child. Fetal Neonatal Ed..

[24] Fanos V., Puddu M., Reali A., Atzei A., Zaffanello M. (2010). Perinatal nutrient restriction reduces nephron endowment increasing renal morbidity in adulthood: A review. Early Hum. Dev..

[25] Dessì A., Ottonello G., Fanos V. (2012). Physiopathology of intrauterine growth retardation: From classic data to metabolomics. J. Matern. Fetal Neonatal Med..

[26] Ju H., Chadha Y., Donovan T., O’Rourke P. (2009). Fetal Macrosomia and pregnancy outcomes. J. Obstet. Gynaecol..

[27] Lawson A.M., Chalmers R.A., Watts R.W. (1976). Urinary organic acids in man. I. Normal patterns. Clin. Chem..

[28] Su T., Xin L., He Y.G., Wei Y., Song Y.X., Li W.W., Wang X.M., He R.Q. (2013). The abnormally high level of uric d-ribose for type-2 diabetics. Prog. Biochem. Biophys..

[29] Fitzhardinge P.M., Steven E.M. (1972). The small for date infant. II. Neurological and intellectual sequelae. Pediatrics.

[30] Hadders-Agra M., Huisjes H.J., Touwen B.C. (1988). Preterm or small for gestational age infants. Neurological and behavioural development at the age of 6 years. Eur. J. Pediatr..

[31] Barberini L., Noto A., Fattuoni C., Grapov D., Casanova A., Fenu G., Gaviano M., Carboni R., Ottonello G., Crisafulli M. (2014). Urinary metabolomics (GC-MS) reveals that low and high birth weight infants share elevated inositol concentrations at birth. J. Matern. Fetal Neonatal Med..

[32] Dessì A., Marincola F.C., Pattumelli M.G., Ciccarelli S., Corbu S., Ossicini C., Fanos V., Agostino R. (2014). Investigation of the **¹**H-NMR based urine metabolomic profiles of IUGR, LGA and AGA newborns on the first day of life. J. Matern. Fetal Neonatal Med..

[33] Dessì A., Fanos V. (2013). Myoinositol: A new marker of intrauterine growth restriction?. J. Obstet. Gynaecol..

[34] Neuberger A. (1981). The metabolism of glycine and serine. Comp. Biochem..

[35] Gannon M.C., Nuttall J.A., Nuttall F.Q. (2002). The metabolic response to ingested glycine. Am. J. Clin. Nutr..

[36] O'Donovan D.J., Fernandes C.J. (2004). Free radicals and diseases in premature infants. Antioxid. Redox Signal..

[37] Saugstad O.D. (2003). Bronchopulmonary dysplasia-oxidative stress and antioxidants. Semin. Neonatol..

[38] Dani C., Cecchi A., Bertini G. (2004). Role of oxidative stress as physiopathologic factor in the preterm infant. Minerva Pediatr..

[39] Njålsson R., Norgren S. (2005). Physiological and pathological aspects of GSH metabolism. Acta Paediatr..

[40] Jain A., Mehta T., Auld P.A., Rodrigues J. (1995). Glutathione metabolism in newborns: Evidence for glutathione deficiency in plasma bronchoaveolar lavage fluid, and lymphocytes in prematures. Pediatr. Pulmonol..

[41] Mabrouk G.M., Jois M., Brosnan J.T. (1998). Cell signalling and the hormonal stimulation of the hepatic glycine cleavage enzyme system by glucagon. Biochem. J..

[42] Yu Y.M., Ryan C.M., Fei Z.W., Lu X.M., Castillo L., Schultz J.T., Tompkins R.G., Young V.R. (2002). Plasma l-5-oxoproline kinetics and whole blood glutathione synthesis rates in severely burned adult humans. Am. J. Physiol. Endocrinol. Metab..

[43] Persaud C., Forrester T., Jackson A. (1996). Urinary excretion of 5-l-oxoproline (pyroglutamic acid) is increased during recovery from severe childhood malnutrition and responds to supplemental glycine. J. Nutr..

[44] Topp H., Fusch G., Schöch G., Fusch C. (2008). Noninvasive markers of oxidative DNA stress, RNA degradation and protein degradation are differentially correlated with resting metabolic rate and energy intake in children and adolescents. Pediatr. Res..

[45] Dzúrik R., Spustová V., Lajdová I. (1993). Inhibition of glucose utilization in isolated rat soleus muscle by pseudouridine: Implications for renal failure. Nephron.

[46] Eriksson L., Johansson E., Kettaneh-Wold N., Trygg J., Wikström C., Wold S. (2013). Multi- and Megavariate Data Analysis.

